# Sirolimus for the treatment of polyposis of the rectal remnant and ileal pouch in four patients with familial adenomatous polyposis: a pilot study

**DOI:** 10.1136/bmjgast-2020-000497

**Published:** 2020-12-29

**Authors:** Victorine H Roos, Bartolomeus J Meijer, Frank G J Kallenberg, Barbara A J Bastiaansen, Lianne Koens, Frederike J Bemelman, Patrick M M Bossuyt, Jarom Heijmans, Gijs van den Brink, Evelien Dekker

**Affiliations:** 1 Department of Gastroenterology and Hepatology, Amsterdam UMC - Location AMC, University of Amsterdam, Cancer Center Amsterdam, Amsterdam, North Holland, The Netherlands; 2 Department of Pathology, Amsterdam UMC - Location AMC, University of Amsterdam, Amsterdam, North Holland, The Netherlands; 3 Department of Internal Medicine, Division of Nephrology, Amsterdam UMC - Location AMC, University of Amsterdam, Amsterdam, North Holland, The Netherlands; 4 Department of Clinical Epidemiology, Biostatistics and Bioinformatics, Amsterdam UMC - Location AMC, University of Amsterdam, Amsterdam, North Holland, The Netherlands; 5 Department of Internal Medicine and Hametology, Amsterdam UMC - Location AMC, University of Amsterdam, Amsterdam, North Holland, The Netherlands; 6 Roche Innovation Center Basel, Roche, Basel, Basel-Stadt, Switzerland

**Keywords:** familial adenomatous polyposis, chemoprevention, adenoma, immunohistochemistry

## Abstract

**Objective:**

After prophylactic colectomy, adenomas continue to develop in the remaining intestine of patients with familial adenomatous polyposis (FAP). There is a lack of standard clinical recommendation for chemoprevention in patients with FAP. Because of promising in vivo studies, the aim of this pilot study was to investigate the safety of sirolimus and its effect on progression of intestinal adenomas.

**Design:**

Patients with FAP with InSiGHT Polyposis Staging System 3 of the retained rectum or pouch received sirolimus for 6 months, dosed at plasma concentration levels of 5–8 µg/L. Primary outcomes were safety and change in marked polyp size. Secondary outcomes were change in number of polyps and effect on proliferation and apoptosis assessed by immunohistochemistry.

**Results:**

Each of the included four patients reported 4 to 18 adverse events (toxicity grades 1–3). One patient prematurely terminated the study because of adverse events. Marked polyp size decreased in 16 (80%)/20 and remained the same in 4 (20%)/20 patients. The number of polyps decreased in all patients (MD −25.75, p=0.13). Three out of four patients showed substantial induction of apoptosis or inhibition of proliferation.

**Conclusion:**

Six months of sirolimus treatment in four patients with FAP showed promising effects especially on the number of polyps in the rectal remnant and ileal pouch, although at the cost of numerous adverse events.

**Trial registration number:**

ClinicalTrials.gov ID NCT03095703.

## Introduction

Familial adenomatous polyposis (FAP) is caused by a mutation in the adenomatous polyposis coli (*APC*) gene and characterised by the development of hundreds to thousands of colorectal adenomas. When left untreated, the risk of developing colorectal cancer is nearly 100% with a mean age at diagnosis of 45 years.^1^ To prevent the development of colorectal cancer, international guidelines recommend a prophylactic colectomy.^2 3^ However, after removal of the colon, adenomas and carcinomas still continue to develop in the remaining large and small intestine. Therefore, lifelong endoscopic surveillance is recommended.

In recent years several drug therapies aiming to decrease polyp burden and potentially delay surgery in patients with FAP have been investigated.^4^ The *APC* mutations that initiate adenoma development enhance epithelial proliferation by activating Wnt-signalling and lead to clonal expansion of the mutated epithelial cell. This epithelial growth process is dependent on increased protein translation driven by activity of mammalian target of rapamycin (mTOR) as part of the mTOR complex 1.^5^ *In vivo* studies in *Apc* mutant mice showed that inhibiting mTORC1 signalling with the mTORC1 inhibitor sirolimus strongly decreased epithelial proliferation and tumour growth in *Apc* mutant adenomas, while not affecting the proliferation of normal intestinal epithelial cells.^5^ Treatment with sirolimus not only decreased intestinal adenoma formation but even led to polyp regression.^6 7^ Moreover, sirolimus increased survival and time to progression to dysplasia.^6 8^

Sirolimus has been widely investigated in human studies as an immunosuppressive agent in patients with renal transplantations. In patients with FAP, only low-dose sirolimus (0.05–0.1 mg/kg) treatment has been studied in two children precolectomy and showed to reduce both the size of duodenal and colonic adenomas as well as the severity in dysplasia.^9^ The aim of our pilot study was to investigate the safety of sirolimus and the effect on the progression of intestinal adenomas in adult patients with FAP with InSiGHT Polyposis Staging System (IPSS) 3 of the retained rectum or pouch, using both clinical as well as molecular outcomes.

## Methods

### Study design and patient population

Inclusion and exclusion criteria of our prospective pilot study are defined in the online supplemental table 1. We included adult patients with classical FAP and a confirmed *APC* mutation having IPSS 3 rectal or pouch polyposis. All patients provided written informed consent, and ethical approval was obtained from the Academical Medical Center institutional review board. Participants who withdrew from the study were not replaced. When these participants had been using sirolimus for at least 1 month, a lower gastrointestinal (LGI) endoscopy was arranged within 6 weeks after withdrawal.

10.1136/bmjgast-2020-000497.supp1 Supplementary data

### Study intervention

Eligible patients received sirolimus treatment (2 mg one time per day) for a duration of 6 months. Sirolimus was provided free of charge by Pfizer BV. After 7 to 10 days sirolimus blood levels were measured. If not within the target range of 5–8 µg/L (based on experiences in the field on renal transplantation in the Academic Medical Center), dosing adjustments were made. In case of dosing adjustments, sirolimus level testing was performed 7 to 10 days hereafter until the target range was achieved. If the sirolimus levels were within the target range, level measurements were performed at 3 months and 6 months. Drug compliance was assessed by pill count review at 3 and 6 months.

### Primary and secondary outcomes

The primary objective was to assess the safety outcomes by analysis of adverse events (by monthly telephone calls), laboratory abnormalities and regular physical examination (during hospital visits at 3 and 6 months). The second primary objective was to evaluate the effect of sirolimus on the size of five marked polyps with an adenomatous appearance. During baseline LGI endoscopy five polyps between 3 and 10 mm were measured (using an open biopsy forceps) and photo documented. Hereafter these polyps were marked using tattoo dye (SPOT) and photographed with ink marking. Six months after the sirolimus treatment, the polyp size was estimated by the endoscopist using an open biopsy forceps. All endoscopies were performed by two experienced endoscopists on dedicated FAP programmes.

Secondary objectives were to evaluate the effect of the sirolimus treatment on the number of polyps (only counting these with an adenomatous aspect on optical diagnosis) and global polyp burden at baseline and 6 months. The global polyp burden was assessed by the endoscopist and valued −2 (much better), −1 (better), 0 (same), 1 (worse) or 2 (much worse) relative to the baseline LGI endoscopy. Furthermore, quality of life was evaluated at baseline, 3 and 6 months using health related quality of life (HRQOL) questionnaires (EORTC QLQ-C30, EORTC QLQ-CR29, SF-36 and EQ-5D-5L).

The size of the marked polyps and total number of polyps were assessed by two independent reviewers (VHR and BJM) scoring the matched still images and videos per patient before and after treatment both blinded for the order. Since the independent reviewers had the same level of experience, the mean size per polyp and mean number of polyps were calculated and presented adjoining the endoscopists review.

Finally, five biopsies of normal appearing mucosa and five biopsies of adenomatous tissue at baseline and after 6 months of sirolimus treatment were taken. Immunohistochemistry was performed in paraffin-embedded slides according to routine methodology. Per biopsy one image at 10× magnification was processed using Olympus BX51 microscope and quantified with ImageJ (V.1.52a, National Institutes of Health). In a blinded manner, per microscopic field, positive-stained epithelial cells were counted and divided by the amount of crypts present at the microscopic field for pS6 (CST #9205) and cleaved caspase 3 (CST #9661). For Ki67 (DAKO, Glostrup) positive staining was quantified as area in ImageJ and divided by haematoxylin positive staining within the same epithelial selected area.

### Statistical analysis

Descriptive statistics were used for the demographic characteristics, adverse events, change in marked polyp size, number of polyps, global polyp burden and HRQOL questionnaire results. SPSS for Windows software (V.21.0, Chicago, Illinois, USA) was used for the analysis.

## Results

### Demographic characteristics of patients

From October 2017 until December 2018, 11 patients were approached for study participation, six patients declined to participate (online supplemental figure 1). Five patients underwent a baseline LGI endoscopy. One of these patients could, despite an IPSS 3, not be included in the study because polyps were too small for marking. Of the four remaining patients, one patient withdrew study participation after 3 months because of adverse events and underwent a LGI endoscopy 2 days after withdrawal.

10.1136/bmjgast-2020-000497.supp2 Supplementary data

All four patients were Caucasian, two were male and median age was 50 (range 42–60) years. Patients 1, 2 and 3 had undergone a total proctocolectomy with ileal pouch-anal anastomosis, while patient 4 had undergone a subtotal colectomy with ileorectal anastomosis. All patients had an *APC* mutation in the premutation cluster region (5′ to 1250). Patients 3 and 4 had a previous history of smoking and none of them had a history of desmoids.

### Clinical outcomes

Four to 16 adverse events per included patient were reported during 6 months of sirolimus treatment ranging from toxicity grade 1 to 2 (online supplemental table 2). Frequencies of adverse events seemed dose-dependent until 3 mg and concentration dependent until 6 µg/L. The events occurred after a median of 59 days (range 2–154). Patient 3 used 2 mg of sirolimus and experienced 18 adverse events during the first 3 months, with toxicity grades varying from 1 to 3, and prematurely terminated participation. The adverse events described were predominantly gastrointestinal disorders (29%), related to study investigations (12%) and skin disorders (12%). One serious adverse event was observed in patient 2: the discovery of a desmoid tumour in the abdominal wall (2.7 × 1.7 × 8.0 cm) located near a scar. One year prior to the start of the study no desmoid tumour was observed on a CT scan, performed 2 months after a redo of the pouch because of a dysfunctional pouch. The family history of the patient was positive for desmoid disease. This patient wished to finish the study and subsequently started sulindac treatment (150 mg two times per day), after which the desmoid tumour showed regression.

During the study period sirolimus dosing varied from 1.5 mg to 4 mg per day and sirolimus levels varied from 2.87 µg/L to 11.0 µg/L (online supplemental figure 2). A median of six (range 3–8) blood tests for sirolimus level testing were needed per patient.

10.1136/bmjgast-2020-000497.supp3 Supplementary data

**Figure 1:**
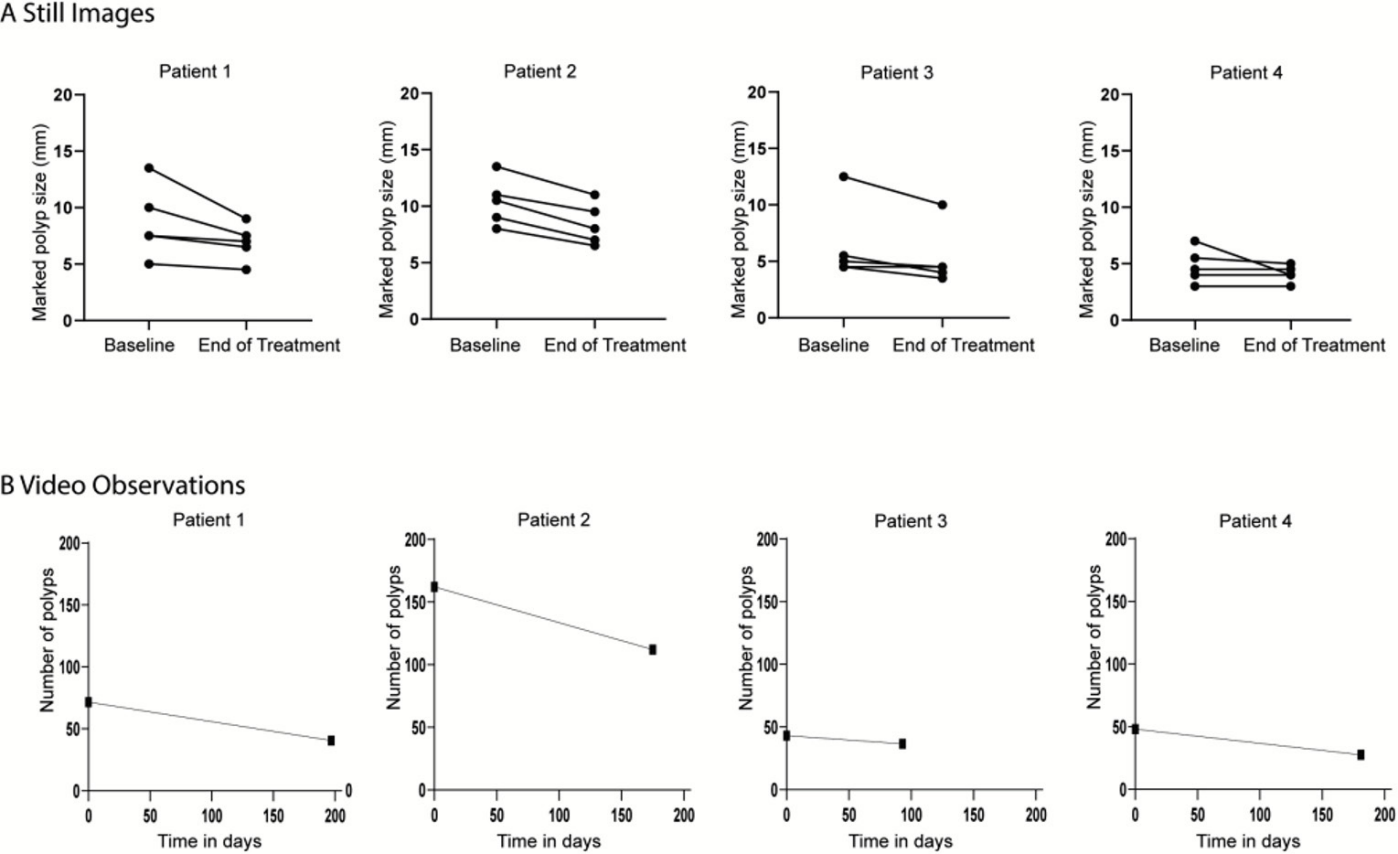
(A) Marked polyp size before and after sirolimus treatment, shown per patient, according to the matched still images assessed by two independent reviewers. (B) Total number of polyps before and after sirolimus treatment, shown per patient, according to the video observations assessed by two independent reviewers blinded for the order.

According to the video observations, all five marked polyps decreased in size in patients 1 and 2, to a lesser extent in patient 3 (4(80%)/5) and patient 4 showed a decrease in only two polyps (figure 1A). These were also shown to a lesser extent in the procedural observations (9 (45%)/20 decrease, 11 (55%)/20 remained the same, online supplemental figure 3A).

10.1136/bmjgast-2020-000497.supp4 Supplementary data

**Figure 2:**
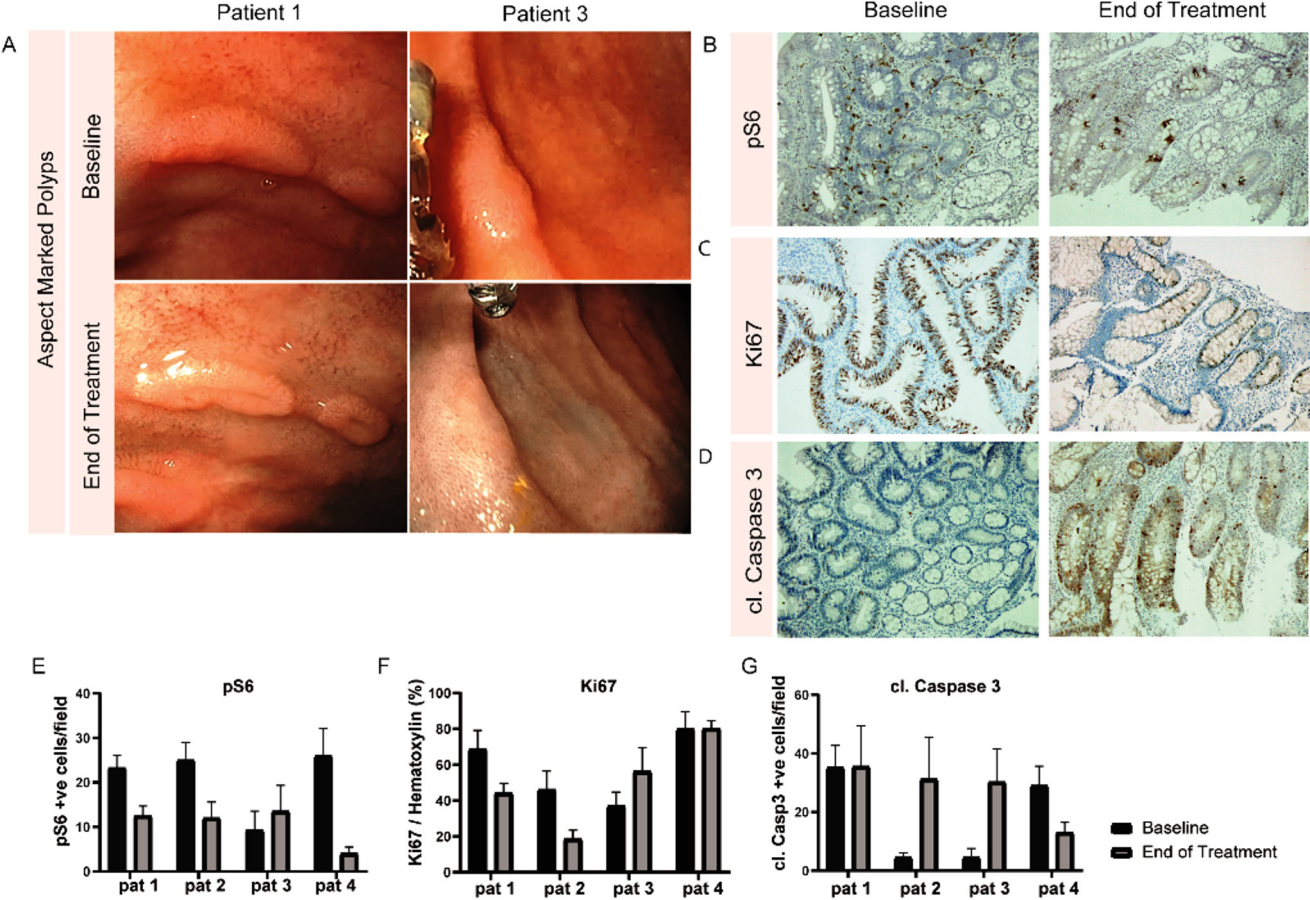
(A) Aspect of adenoma before and after sirolimus treatment, showing a central depression in the adenoma in patient 1 and demonstrating a more flattening aspect of an adenoma after treatment in patient 3. (B–D) Representative image of phosphorylated S6, Ki67 and cleaved caspase 3 throughout adenomatous tissue, shown for patient 2. (E) Left panel: quantification of pS6 positive cells per 10× microscopic field. Middle panel: quantification of Ki67 relative to haematoxylin staining for selected epithelium within a 10× microscopic field. Right panel: quantification of cleaved caspase 3 positive cells per 10× microscopic field.

All patients showed a decrease in the total number of polyps both in the video observations (median difference −25.75, p=0.13, figure 1B) as in the procedural observations (median difference −12.50, p=0.13, online supplemental figure 3B). The global polyp burden had improved in patients 1, 2 and 3 and had not changed in patient 4. A more detailed overview of the global polyp burden assessed by the endoscopist is shown in the online supplemental table 3. Interestingly, the aspect of several polyps showed a very distinct morphological change after sirolimus treatment demonstrating a more flattened appearance with a central dimple. The morphological change appeared to suggest polyp regression (figure 2A).

Each patient reported 4 to 18 adverse events (toxicity grades 1–3). One patient prematurely terminated the study because of adverse events. Size marked polyps decreased in 16 (80%)/20 and remained the same in 4 (20%)/20. The total number of polyps decreased in all patients. Three out of four patients showed substantial induction of apoptosis or inhibition of proliferation.

The HRQOL questionnaires reported increase in urinary frequency, blood and mucus in the stool, dysgeusia, flatulence, faecal incontinence, stool frequency, decrease in sexual function, increase in fatigue, dyspnoea, insomnia and diarrhoea (online supplemental figure 4). Furthermore, the global health status had decreased in patients 2, 3 and 4.

10.1136/bmjgast-2020-000497.supp5 Supplementary data

### Immunohistochemistry outcomes

In all patients, adenoma biopsies were evaluated by an experienced pathologist (LK) before and after treatment and demonstrated low-grade dysplasia. As expected, phosphorylation of mTOR downstream signalling target ribosomal protein S6 was decreased in adenomatous tissue compared with adjacent healthy biopsies at baseline as detected with a phosphorylation-specific S6 antibody. After treatment with sirolimus, phosphorylation of the ribosomal S6 protein decreased in the adenomatous tissue in all four patients, suggesting that the sirolimus dose was adequate for mTORC1 target engagement and inhibition (figure 2B, E). The effect of mTORC1 inhibition on proliferation was assessed by immunostaining of Ki67 in the intestinal epithelium. Changes in proliferation were heterogeneous throughout the biopsies of all patients. Nevertheless, the largest and most consistent reduction of proliferation was found in patients 1 and 2 corresponding to the decrease in adenoma numbers (figure 2C, E). Furthermore, apoptosis was addressed by cleaved caspase 3 staining and showed most apoptosis in patients 2 and 3 (figure 2D, E).

## Discussion

The current study is the first to evaluate the effect of sirolimus treatment in adults with FAP having IPSS 3 staged rectal remnant or pouch polyposis using both clinical as well as molecular outcomes. Being a pilot study, power was limited and the study had no comparator group. Nevertheless, outcomes were well defined and prespecified and assessment of outcomes was blinded. Inline with the case report of Yuksekkaya *et al* we found that sirolimus reduced the number of polyps, could improve the global polyp burden and decreased the marked polyp size in some patients, in contrast to expected stable or progressive disease.^9^ Although sirolimus dosing was in comparable therapeutic range, patients in our study suffered from considerably more adverse events. According to the literature in renal transplant patients, adverse events are influenced by sirolimus levels of >15 µg/L.^10^ In this study, measured concentrations were lower than 11.0 µg/L and only showed dose and concentration dependency up to a certain level. Furthermore, the majority of the adverse events were transient in nature while administration of sirolimus was continued, suggesting no direct dose or concentration relationship using the target range of 5–8 µg/L. It is unknown if the desmoid tumour that occurred was already present at the start of the study. However, the recent operation and positive family history suggest this to be a result of the nature of the underlying disease.

Immunohistochemistry showed an decreased expression of S6 phosphorylation in adenomatous tissue in all four patients, comparable to the studies in an *Apc* mutant mouse model of FAP.^2^ The reduction of phosphorylated S6 during sirolimus treatment suggests that the target plasma concentration resulted in adequate target engagement and mTORC1 inhibition in the intestinal adenomas. Effects on proliferation however were heterogeneous. Patients 1 and 2 showed more promising results on adenoma numbers and proliferation rate than patients 3 and 4. Patient 3 had stopped sirolimus treatment for several days before colonoscopy, potentially explaining the lack of impaired proliferation. Patient 4 did not seem to benefit from sirolimus treatment. Although we observed increased apoptotic cell death in adenomas, this varied per patient and may have been influenced by sample variance.

In conclusion, maintenance therapy with sirolimus at a target plasma concentration of 5–8 µg/L is sufficient to inhibit mTORC1 in intestinal adenomas, reduces the number of polyps and leads to a reduction in proliferation. This recapitulates the effect of sirolimus treatment observed in *Apc* mutant mice. To overcome the relatively poor tolerability of sirolimus using this therapeutic target range in these patients, strategies to reduce side effects, such as lowering the sirolimus target range in a phase IIb study, use of an mTOR inhibitor with better tolerability or ideally the development of a gut-targeted inhibitor of mTOR could be considered. Nevertheless, our promising results suggest that these efforts could lead to a new chemopreventive approach for patients with FAP.
