# Supplementary material for: Sirolimus for the treatment of polyposis of the rectal remnant and ileal pouch in four patients with familial adenomatous polyposis: a pilot study
Source: BMJ Open Gastroenterol. 2020 Dec 29;7(1):e000497. doi: 10.1136/bmjgast-2020-000497 (PMC7778746; doi:10.1136/bmjgast-2020-000497)
Supplement: Supplementary data [file bmjgast-2020-000497supp001.pdf]

Supplementary Materials Tables

Table 1\ In- and Exclusion criteria

**Inclusion criteria**
In order to be eligible to participate in this study, a subject must meet all of the following criteria:<br>- ≥ 18 years<br>- A genetically confirmed APC mutation<br>- Classical FAP phenotype (100-1000 colorectal adenomatous polyps)<br>- Subtotal colectomy with ileorectal anastomosis (IRA) or total colectomy with ileo-anal pouch anastomosis (IPAA)<br>- Severe rectal or pouch polyposis, defined as having >25 polyps amenable to complete removal<br>- Fertile patients must use effective contraception during study treatment and until 12 weeks after study treatment
**Exclusion criteria**
A potential subject who meets any of the following criteria will be excluded from participation in this study:<br><br><u>General exclusion criteria:</u><br>- Inability to give informed consent<br>- Participation in another interventional clinical trial<br><br><u>Medical history:</u><br>- Subjects who are pregnant or breast-feeding, proved with a negative pregnancy test if female of child-bearing potential<br>- Prior pelvic irradiation<br>- Invasive malignancy in the past 5 years<br>- Subjects who are HIV positive<br>- Subjects with severe systemic infections, current or within 2 weeks prior to study start<br>- Subjects with known severe restrictive or obstructive pulmonary disorders<br>- Known sucrase insufficiency, isomaltase insufficiency, fructose intolerance, glucose malabsorption, galactose malabsorption, galactose intolerance or Lapp-lactase deficiency<br>- History of pulmonary embolism or deep venous thrombosis<br>- Major surgery less than or equal to 2 weeks prior to enrollment or any planned surgery within treatment period<br>- Active post-operative complication, e.g. infection, delayed wound healing<br><br><u>Co-medication:</u><br>- History of hypersensitivity to sirolimus or its excipients or to drugs of similar chemical classes<br>- Regular NSAID use (defined as more than twice a week for 4 consecutive weeks) within 3 months prior to baseline<br>- Use of other FAP directed drug therapies (accepted if discontinued 3 months prior to start of the study)<br>- Subjects requiring systemic anticoagulation<br>- Co-medication that could interact with sirolimus: Cyclosporine, IL-2-receptor antibodies, Calcineurine inhibitors, HMG-CoA-reductase inhibitors, fibrates, CYP3A4-inhibitors, CYP3A4-inductors, ACE-inhibitors, cisapride, metoclopramide, Pgp inhibitors<br>- Use of grapefruit juice<br>- Use of attenuated vaccins<br><br><u>Lab results:</u><br>Abnormal laboratory results (assessed within 14 days prior to start of study drug):<br>- Significant abnormalities in hepatic function<br>o ALAT, ASAT, GGT, AF, LDH: > 1.5 times ULN<br>- Significant hematologic abnormalities<br>o Hemoglobin: <7.0 mmol/L<br>o Thrombocytes: <100 10E9/L

    - Leukocytes: <4.0 10E9/L
- Increased fasting serum cholesterol or triglyceride (whether or not on lipid-lowering therapy)
    - Serum cholesterol: >7.8 mmol/L
    - Serum triglycerides: >4.5 mmol/L
- Increased glucose (venous, fasting): >6.4 mmol/L
- Electrolyte abnormalities
    - Total serum calcium (corrected for albumin): <2.0 mmol/L
    - Potassium: <3.0 mmol/L
    - Phosphate: <1.3 mmol/L
    - Calculated glomerular filtration rate (GFR) less than 40 mL/min/1.73m2 using the simplified Modification of Diet in Renal Disease (MDRD) formula
- Urine specimen:
    - Spot urine protein to creatinine ratio (UPr/Cr) greater than or equal to 0.5

Table 2\ Overview (Serious) Adverse Events

CTCAE System organ group	Patient	Condition	Toxicity grade (1-5)	Outcome	Duration in days	Relationship to study drug	Actions taken with study drug	Treatment provided
Cardiac disorders	3*	Tachycardia	1	Recovered/ resolved	1	Probably related	None	None
Gastrointestinal disorders	1	Frequent stools	1	Recovered/ resolved	3	Possibly related	None	None
		Bloated feeling	1	Recovered/ resolved	3	Possibly related	None	None
		Increased appetite	1	Recovered/ resolved	62	Possibly related	None	None
		Loss of appetite	1	Recovered/ resolved	43	Not related	None	None
		Diarrhea	2	Recovered/ resolved	25	Possibly related	None	None
		Dry mouth	1	Recovered/ resolved	90	Not related	None	None
	2	Frequent stools	1	Recovered/ resolved	194	Possibly related	None	None
		Mouth ulcers	2	Recovered/ resolved	151	Probably related	None	None
		Diarrhea	1	Recovered/ resolved	1	Possibly related	None	None
		Diarrhea	2	Recovered/ resolved	1	Possibly related	None	None
		Diarrhea	1	Recovered/ resolved	2	Possibly related	None	Medication
	3*	Diarrhea	3	Recovered/ resolved	5	Possibly related	None	None
		Loss of appetite	1	Not recovered/ resolved		Possibly related	None	None
	4	Dental root caries	2	Recovered/ resolved	7	Not related	None	Medication
General disorders	2	Fatigue	1	Recovered/ resolved	64	Possibly related	None	None
	3*	Cold/ dry extremities	1	Not recovered/ resolved		Possibly related	None	None
		Edema limbs	2	Not recovered/ resolved		Probably related	None	None
Infections and infestations	1	Common cold	1	Recovered/ resolved	90	Possibly related	None	None
		Blepharitis	2	Recovered/ resolved	2	Possibly related	None	None
	3*	Common cold	1	Recovered/ resolved	47	Possibly related	None	None
	4	Flu like symptoms	1	Recovered/ resolved	3	Possibly related	None	Medication
Injury, poisoning and procedural complications	3*	Delayed wound healing	1	Recovered/ resolved	30	Probably related	None	None
		Delayed wound healing	1	Recovered/ resolved	30	Probably related	None	None

Investigations	1	High cholesterol	1	Recovered/ resolved	105	Probably related	None	None
	2	Cholesterol increase	1	Not recovered/ resolved		Probably related	None	None
	3*	Weight loss	1	Recovered/ resolved	28	Possibly related	None	None
		Cholesterol increase	1	Recovered/ resolved	28	Probably related	None	Other
		Alkaline phosphatase increase	1	Recovered/ resolved	28	Probably related	None	Other
		lactate dehydrogenase increase	1	Not recovered/ resolved		Probably related	None	Other
Metabolism and nutrition disorders	1	Hypertriglyceridemia	1	Recovered/ resolved	105	Probably related	None	None
	3*	Hypertriglyceridemia	1	Recovered/ resolved	28	Probably related	None	Other
Musculoskeletal and connective tissue disorders	1	Muscle ache	1	Recovered/ resolved	122	Not related	None	None
	2	Muscle strain	1	Recovered/ resolved	14	Possibly related	None	None
		Joint range of motion decrease	1	Recovered/ resolved	1	Possibly related	None	None
	3*	Joint pain	2	Not recovered/ resolved		Not related	None	None
	4	Knee pain	2	Recovered/ resolved	61	Not related	None	Medication
Nervous system disorders	2	Headache	1	Recovered/ resolved	2	Possibly related	None	Medication
		Headache	1	Recovered/ resolved	7	Possibly related	None	None
		Dysgeusia	2	Recovered/ resolved	126	Possibly related	None	None
	3*	Dysgeusia	2	Recovered/ resolved	77	Possibly related	None	None
		Headache	1	Recovered/ resolved	17	Possibly related	None	None
Respiratory, thoracic and mediastinal disorders	3*	Dyspnoea	1	Recovered/ resolved	23	Possibly related	None	None
Skin and subcutaneous tissue disorders	2	Acne face	1	Recovered/ resolved	4	Possibly related	None	None
		Anal discomfort	1	Recovered/ resolved	185	Not related	None	None
		Desmoïd #	2	Not recovered/ resolved		Possibly related	None	Medication
		Acne thighs and face	1	Recovered/ resolved	175	Probably related	None	None
	3*	Skin papules	1	Not recovered/ resolved		Possibly related	None	None

	4	Sebaceous cysts increase	2	Not recovered/ resolved		Possibly related	None	Medication

* Primarily terminated the study.
\# Serious adverse event defined as an important medical event.

Table 3\ Polyp burden assessed during the procedure by the endoscopist

		Number of polyps at baseline			Number of polyps at 6 months		
**Study subject**	**Polyp size**	Afferent loop	Ileal pouch	Rectal cuff	Afferent loop	Ileal pouch	Rectal cuff
**Patient 1**	1-4mm	1	25	5	0	28	2
	5-10mm	0	10	0	0	2	0
	>10mm	0	5	0	0	0	0
	**Total**	**1**	**40**	**5**	**0**	**30**	**2**
**Patient 2**	1-4mm	20	150	0	10	100	0
	5-10mm	0	15	0	0	15	0
	>10mm	0	2	0	0	2	0
	**Total**	**20**	**167**	**0**	**10**	**117**	**0**
**Patient 3**	1-4mm	0	30	7	0	19	7
	5-10mm	0	0	1	0	1	1
	>10mm	0	1	0	0	0	0
	**Total**	**0**	**31**	**8**	**0**	**20**	**8**
**Study subject**	**Polyp size**	Above the rectal anastomosis	NA	Retained rectum	Above the rectal anastomosis	NA	Retained rectum
**Patient 4**	1-4mm	0	NA	50	0	NA	39
	5-10mm	0	NA	1	0	NA	1
	>10mm	0	NA	0	0	NA	0
	**Total**	**0**	**NA**	**51**	**0**	**NA**	**40**
