# Supplementary figures and images for: Sirolimus for the treatment of polyposis of the rectal remnant and ileal pouch in four patients with familial adenomatous polyposis: a pilot study
Source: BMJ Open Gastroenterol. 2020 Dec 29;7(1):e000497. doi: 10.1136/bmjgast-2020-000497 (PMC7778746; doi:10.1136/bmjgast-2020-000497)

Figure 1 Flowchart

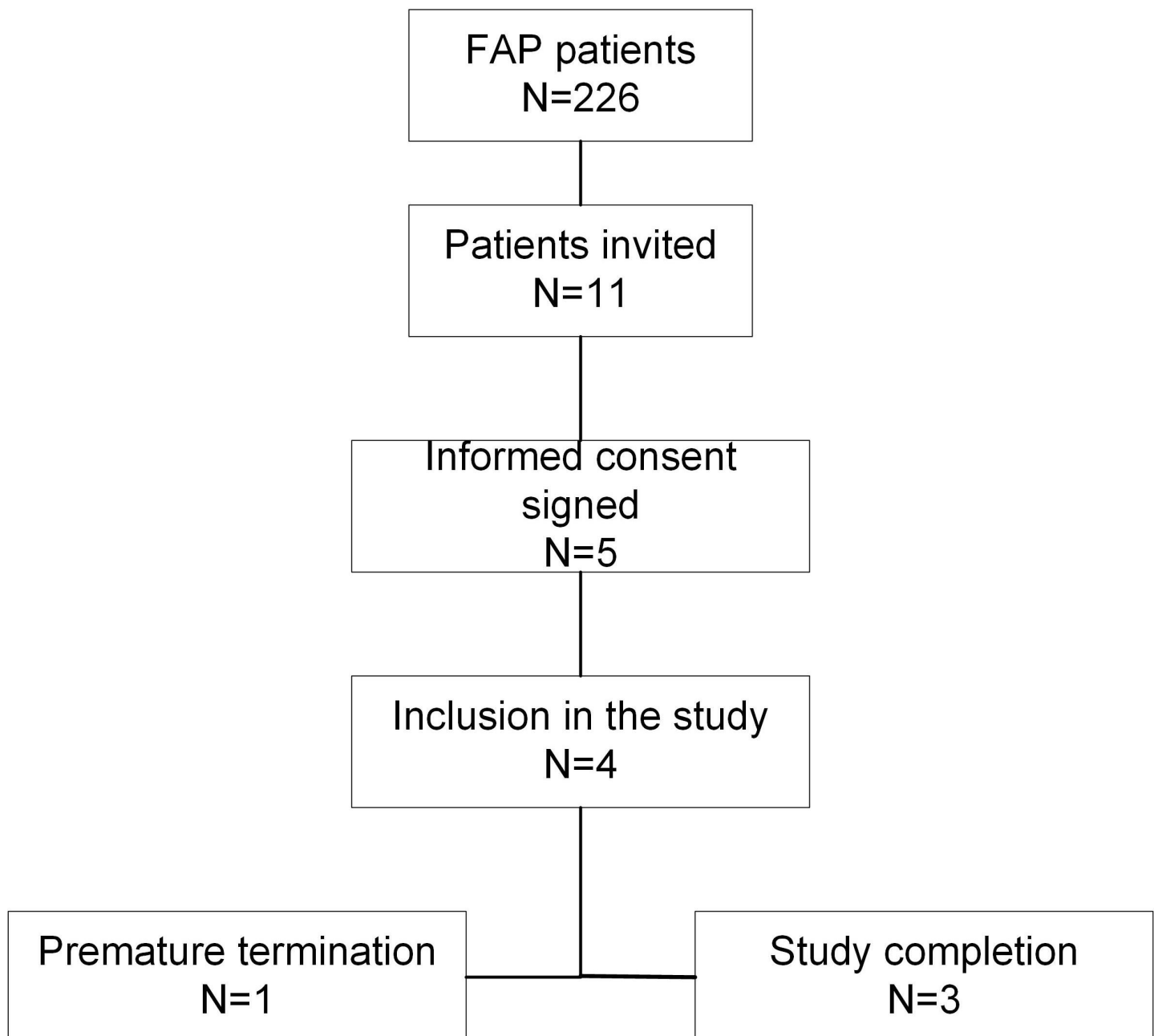

Supplement: Supplementary data [file bmjgast-2020-000497supp002.pdf]

Figure 2 Sirolimus data

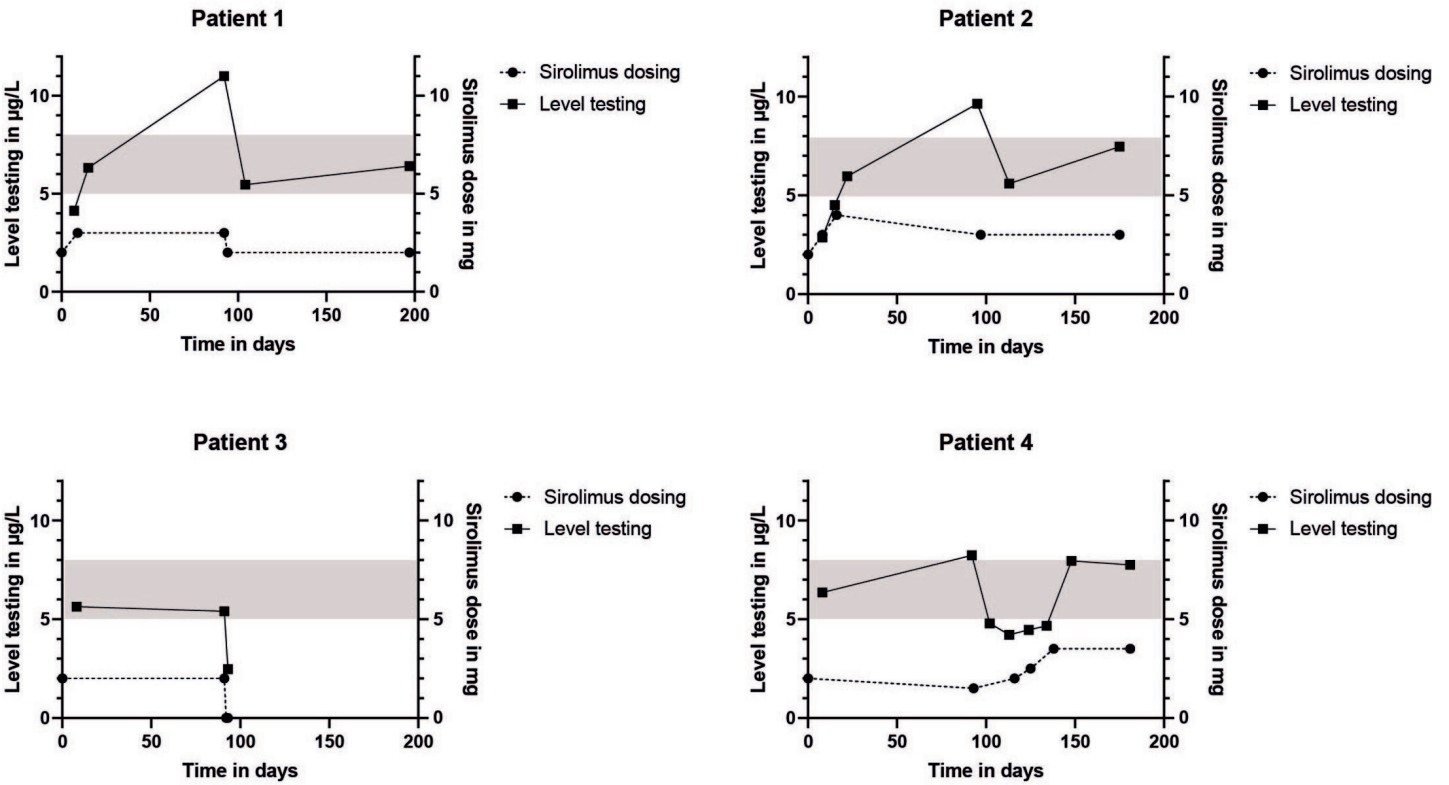

Supplement: Supplementary data [file bmjgast-2020-000497supp003.pdf]

Figure 3 Procedural observations

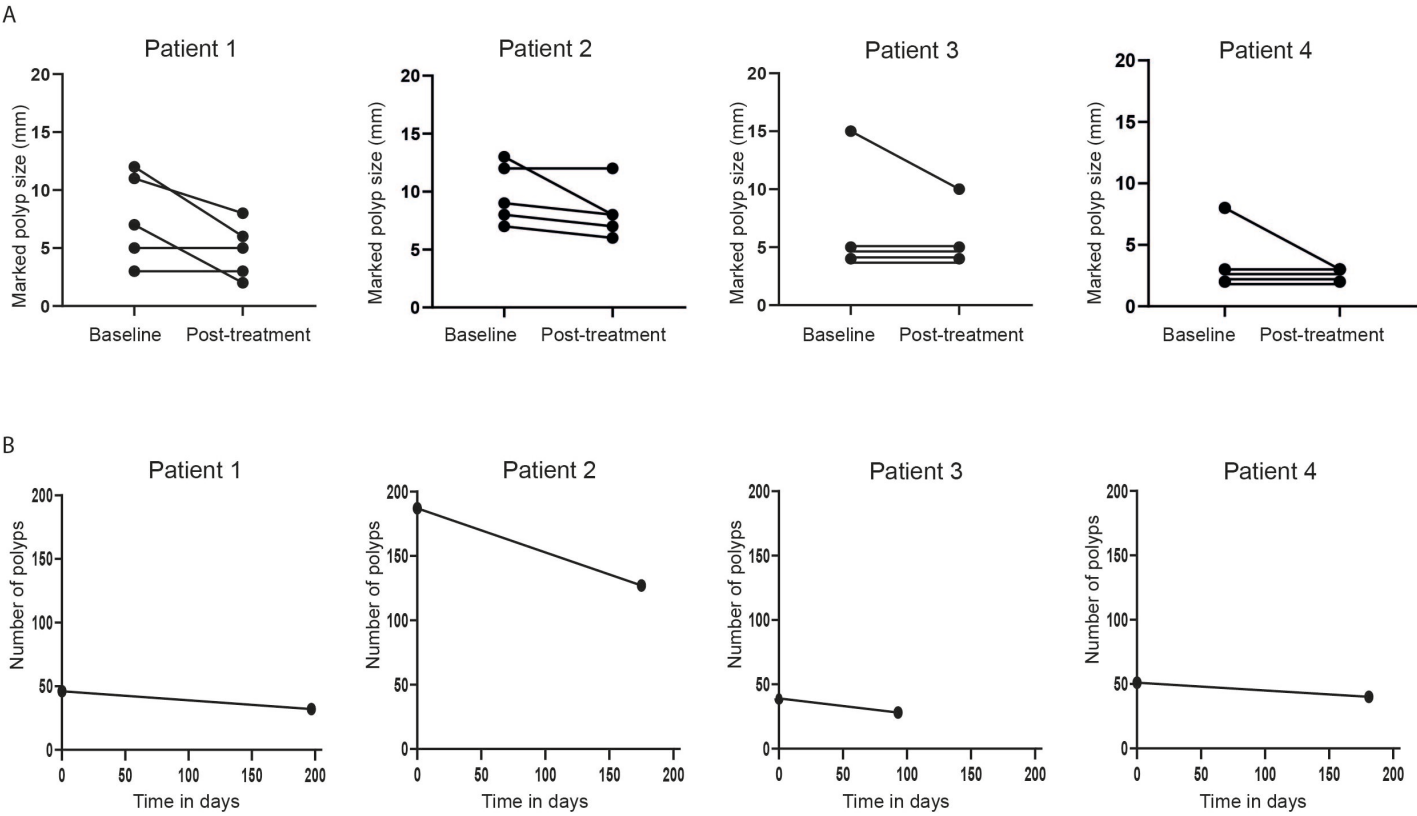

Supplement: Supplementary data [file bmjgast-2020-000497supp004.pdf]

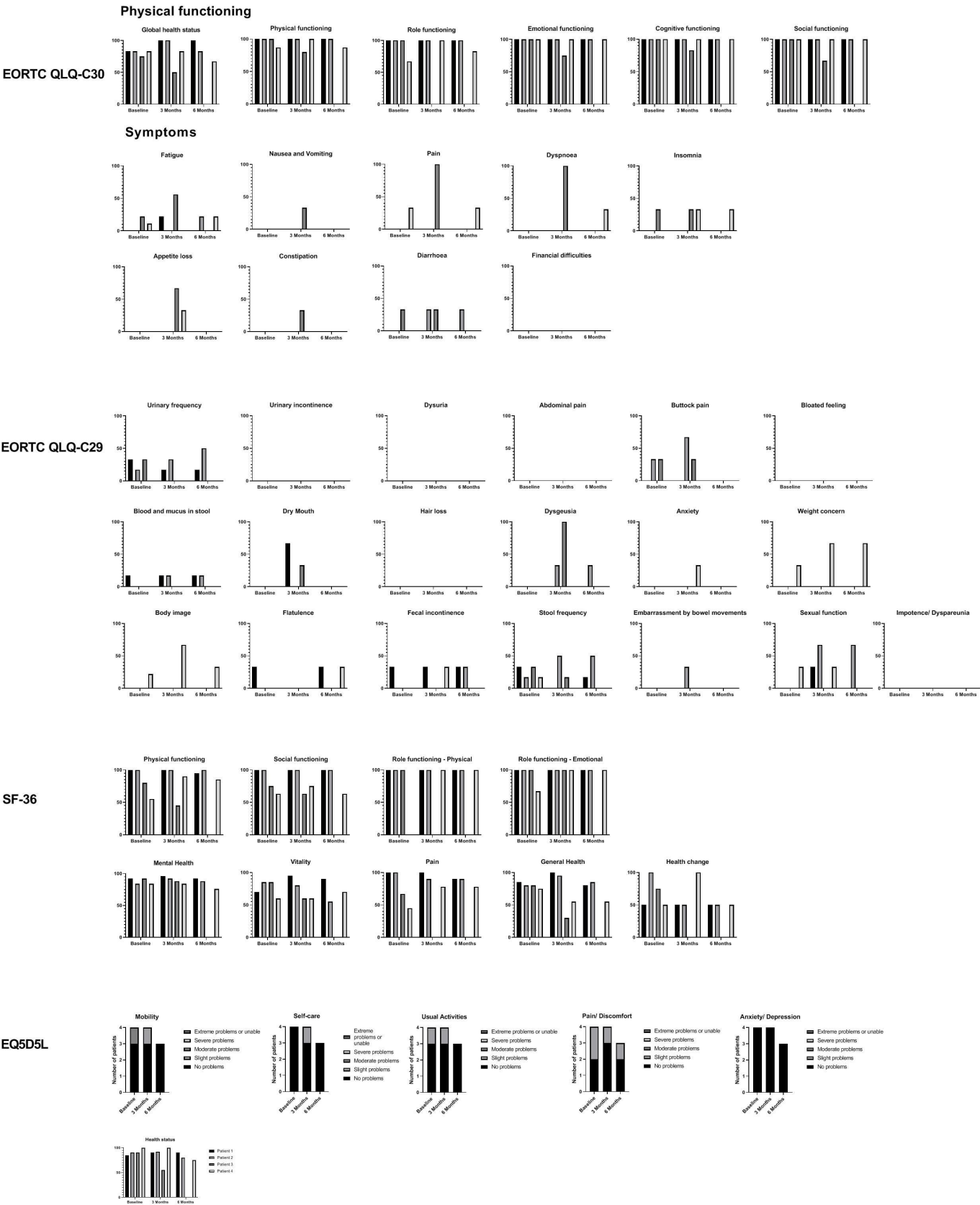

Supplement: Supplementary data [file bmjgast-2020-000497supp005.pdf]
